# Supplementary material for: Genome-Wide Identification and Characterization of Circular RNAs during Skeletal Muscle Development in Meat Rabbits
Source: Animals (Basel). 2022 Aug 27;12(17):2208. doi: 10.3390/ani12172208 (PMC9454498; doi:10.3390/ani12172208)
Supplement: Supplementary file 1 [file animals-12-02208-s001.zip › Table S1.pdf]

Table S1 Summary of sequencing data

Sample	Library type	Read number	Q20	Q30	GC content	Uniquely mapped	Total mapped
D0_1	Whole-transcriptome	94250288	98.19%	94.89%	56%	68.28%	90.66%
D0_2	Whole-transcriptome	83258044	98.00%	94.59%	58%	61.35%	88.24%
D0_3	Whole-transcriptome	87609022	98.18%	94.94%	58%	67.13%	89.36%
D35_1	Whole-transcriptome	115518932	98.40%	95.48%	55%	67.54%	91.36%
D35_2	Whole-transcriptome	86309418	98.29%	95.22%	52%	77.09%	91.79%
D35_3	Whole-transcriptome	112002128	98.43%	95.59%	51%	77.92%	92.24%
D70_1	Whole-transcriptome	98021746	98.31%	95.30%	57%	63.66%	90.48%
D70_2	Whole-transcriptome	87020102	98.27%	95.19%	58%	64.81%	89.25%
D70_3	Whole-transcriptome	86993306	98.47%	95.66%	59%	57.12%	89.51%
Pooled sample	CircRNA-seq	104185248	97.03%	92.6%	65%	74.72%	81.71%
